# Drug‐Coated Balloons Versus Drug‐Eluting Stents or Plain Old Balloon Angioplasty: A Long‐Term in‐Stent Restenosis Study

**DOI:** 10.1161/JAHA.124.036839

**Published:** 2024-11-22

**Authors:** Sacharias von Koch, Mikael Zhou, Hans Christian Rosén, Sammy Zwackman, Juliane Jurga, Per Grimfjärd, Matthias Götberg, Moman A. Mohammad, David Erlinge

**Affiliations:** ^1^ Department of Cardiology, Clinical Sciences Lund University, Skåne University Hospital Lund Sweden; ^2^ Department of Health, Medicine and Caring Sciences and Department of Cardiology Linköping University Linköping Sweden; ^3^ Division of Cardiology, Department of Medicine Karolinska Institute and Karolinska University Hospital Stockholm Sweden; ^4^ Department of Internal Medicine Västmanlands Hospital Västerås Sweden

**Keywords:** drug‐coated balloon, drug‐eluting stent, in‐stent restenosis, plain old balloon angioplasty, target‐lesion revascularization, Percutaneous Coronary Intervention

## Abstract

**Background:**

Balloon angioplasty with drug‐coated balloons (DCBs) is frequently used during percutaneous coronary intervention for in‐stent restenosis. Despite its frequent use, there is a lack of long‐term data on the efficacy of DCB angioplasty. We conducted an investigation on the long‐term efficacy outcome of in‐stent restenosis, comparing DCBs, drug‐eluting stents, and plain old balloon angioplasty.

**Methods and Results:**

We conducted a nationwide analysis from the SCAAR (Swedish Coronary Angiography and Angioplasty Registry) including in‐stent restenosis lesions undergoing coronary angiography between June 11, 2013, and January 14, 2022. The primary outcome of this study was target‐lesion revascularization within a 5‐year follow‐up. Secondary outcomes included all‐cause death, cardiovascular death, myocardial infarction, and any percutaneous coronary intervention. The outcomes were analyzed using a multivariable Cox proportional hazard model or Poisson regression, as appropriate. A total of 10 561 lesions from 9062 patients were included. Compared with plain old balloon angioplasty, the use of DCB angioplasty was associated with less target‐lesion revascularization (risk ratio, 0.69 [95% CI, 0.57–0.82]), all‐cause death (risk ratio, 0.72 [95% CI, 0.59–0.88]), and cardiovascular death (hazard ratio [HR], 0.59 [95% CI, 0.45–0.78]). No difference was observed for myocardial infarction or any percutaneous coronary intervention. Compared with drug‐eluting stents, the use of DCBs was associated with higher rates of target‐lesion revascularization (HR, 1.20 [95% CI, 1.06–1.37]). No difference was observed for all‐cause death, cardiovascular death, myocardial infarction, or any percutaneous coronary intervention.

**Conclusions:**

In this long‐term nationwide analysis, the use of DCB angioplasty showed superior outcomes compared with plain old balloon angioplasty within 5 years but higher rates of repeat revascularizations compared with drug‐eluting stents.

**Nonstandard Abbreviations and Acronyms**
AGENT IDE: A Clinical Trial to Assess the Agent Paclitaxel Coated PTCA Balloon Catheter for the Treatment of Subjects With In‐Stent Restenosis
BIOLUX‐RCT: Angiographic and Clinical Performance of a Paclitaxel‐Coated Balloon Compared to a Second‐Generation Sirolimus‐Eluting Stent in Patients With In‐Stent Restenosis
BMS: bare‐metal stent
DARE: A Randomized Comparison of Paclitaxel‐Eluting Balloon Versus Everolimus‐Eluting Stent for the Treatment of Any In‐Stent Restenosis
DCB: drug‐coated balloon
DAEADALUS: Paclitaxel‐Coated Balloon Angioplasty Versus Drug‐Eluting Stenting for the Treatment of Coronary In‐Stent Restenosis
DES: drug‐eluting stent
ISAR DESIRE 3: Coronary Artery Restenosis Treatment With Plain Balloon, Drug‐Coated Balloon, or Drug‐Eluting Stent
ISR: in‐stent restenosis
POBA: plain old balloon angioplasty
RIBS IV/V: Comparison of the Efficacy of Everolimus‐Eluting Stents Versus Drug‐Eluting Balloons in Patients With In‐Stent Restenosis
SCAAR: Swedish Coronary Angiography and Angioplasty Registry
TLR: target‐lesion revascularization

**Clinical Perspective**

**What Is New?**

- Most randomized trials investigating drug‐coated balloons (DCBs) for in‐stent restenosis have follow‐up times ranging between 1 year and 3 years; the follow‐up time of this study was 5 years.
- In this study, 10 561 in‐stent restenosis lesions from 9062 patients were included, the study population allows enough power to assess clinical outcomes such as target‐lesion revascularization and death.
- Most previous trials have focused either on bare‐metal stents–in‐stent restenosis or drug‐eluting stents (DESs)–in‐stent restenosis and used a specific type of DCB restricting the generalizability of these studies; we included any underlying stent and different types of DCBs.

**What Are the Clinical Implications?**

- In this study comparing DCBs, DESs, and plain old balloon angioplasty, our findings indicate superior outcome for DCB‐treated lesions compared with plain old balloon angioplasty–treated lesions.
- While DCBs demonstrated higher rates of target‐lesion revascularization than DESs, the lack of mortality differences indicates that DCBs are a viable option, particularly for patients for whom DESs might not be suitable, but that DESs should be considered as the first‐line strategy.

Percutaneous coronary intervention (PCI) using coronary artery stenting is the standard treatment for managing obstructive coronary artery disease, effectively overcoming the limitations associated with plain old balloon angioplasty (POBA).^1^ The implementation of drug‐eluting stents (DESs) has reduced rates of in‐stent restenosis (ISR) compared with bare‐metal stents (BMSs).^2^, ^3^, ^4^ Despite substantial progress in treatment techniques, ISR continues to pose a significant challenge, accounting for about 10% of all PCI cases.^5^ Furthermore, ISR is associated with adverse outcomes compared with de‐novo lesions.^6^ Considering this, choosing an effective treatment strategy for patients with ISR is essential for ensuring optimal outcomes. For patients with ISR, repeat stenting with DESs has been associated with lower rates of target‐lesion revascularization (TLR) when compared with POBA alone.^7^ In recent years, DCB angioplasty) has emerged as an alternative treatment strategy to DESs. The ISAR‐DESIRE 3 trial showed that DCBs were noninferior to DESs and superior to POBA in terms of diameter stenosis on follow‐up angiography after 6 to 8 months.^8^ In line with the class 1, level A recommendation by European guidelines supporting the use of either DESs or DCBs for the management of ISR, there has been a notable increase in DCB penetration in clinical practice where DCBs are used in 7.2% of all coronary interventions.^9^ However, data on the long‐term efficacy of DCBs for ISR remain scarce. To date, most of the trials on DCBs have follow‐up inferior to 3 years. In addition to short follow‐up periods and limited study populations, prior randomized trials and observational studies have yielded inconsistent results on the efficacy of DCBs with regards to rates of TLR, where in some trials DCBs have been associated with a higher TLR rate compared with DESs.^10^, ^11^, ^12^, ^13^ Other randomized trials and observational studies have found no difference in TLR.^14^, ^15^, ^16^, ^17^, ^18^

Given this, large high‐quality observational data on this topic are warranted. In this nationwide lesion and patient‐level analysis, we sought to compare the long‐term outcomes of DCBs, DESs, and POBA for ISR.

## Methods

### Data Source

We conducted a nationwide lesion‐level analysis using the SCAAR (Swedish Coronary Angiography and Angioplasty Registry).^19^ SCAAR include data from all patients in Sweden undergoing coronary angiography or intervention in any of the 29 PCI centers providing acute cardiac care. The SCAAR is a nationwide quality registry, and all patients are informed about the inclusion in the registry and the option to opt out of the registry. No written consent is necessary. The registry includes extensive information on lesion characteristics (eg, lesion location, lesion complexity), patient characteristics (eg, risk factors, age, sex) and procedural information (eg, stent type, stent sizing). The Swedish Prescribed Drugs registry was used to collect data on dispensed medical therapies 90 days before to 14 days after PCI. We cannot make data available, but interested researchers can contact us and we may help with presenting aggregated data.

### Study Design, Study Population, and Outcomes

The study was approved by the Swedish Ethical Review Authority (Dnr 2023‐00201‐01), and research was carried out in accordance with appropriate ethical guidelines.

**Figure 1:**
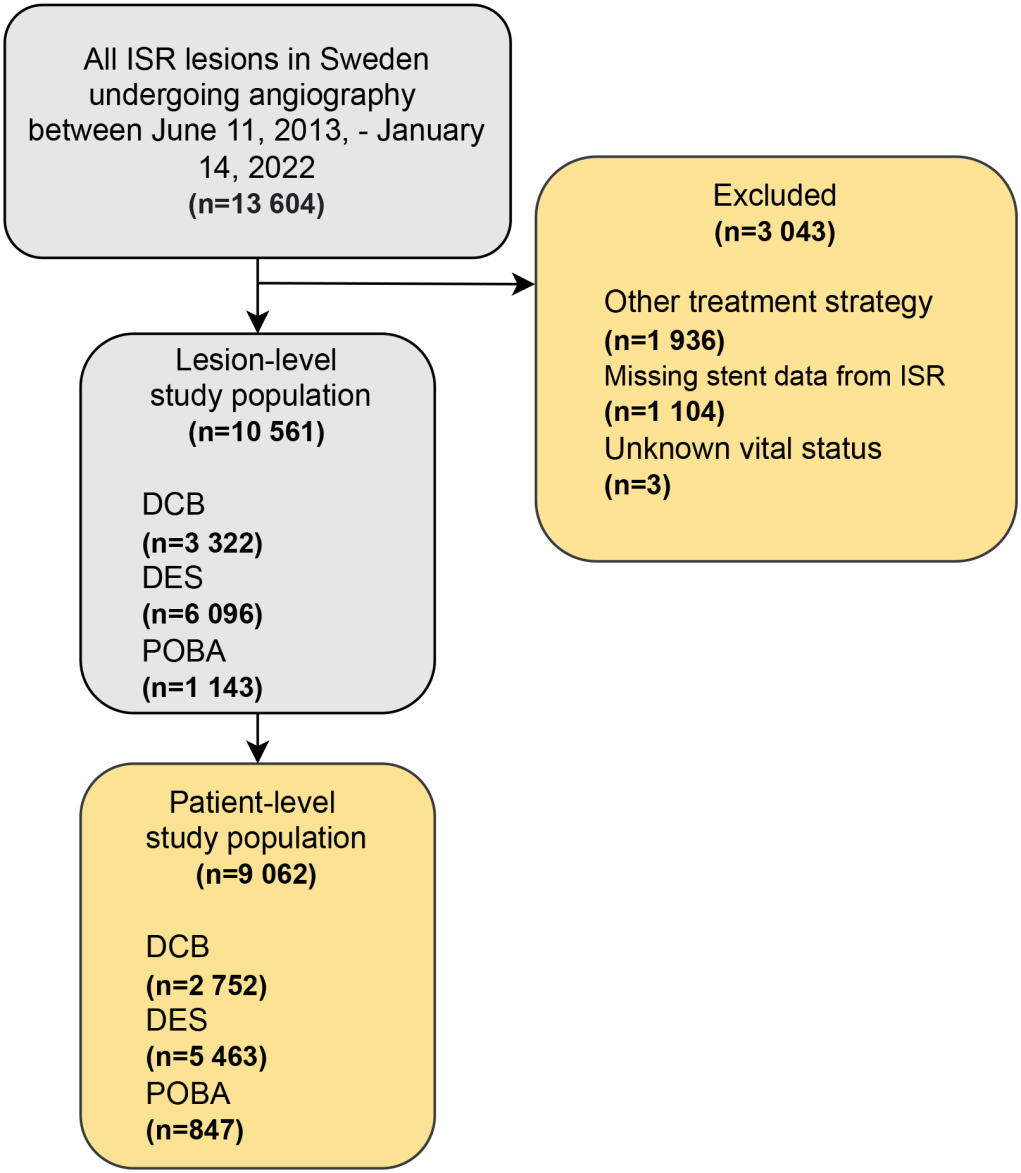
Flowchart. Flowchart illustrating eligible lesions and patients. The final study population consisted of 10 561 lesions from 9062 patients. DCB indicates drug‐coated balloon; DES, drug‐eluting stent; ISR, in‐stent restenosis; and POBA, plain old balloon angioplasty.

A flowchart including exclusion and inclusion criteria is presented in Figure 1. The inclusion time of this study was between June 11, 2013, and January 14, 2022. Using the SCAAR, we included all lesions presenting with ISR on angiography. Exclusion criteria comprised lesions treated with BMSs, first‐generation DESs, degradable scaffolds, DESs used ≤10 times in the registry during the study period, coronary artery bypass graft surgery, or a conservative strategy of medical therapy only. Eligible lesions were stratified into 3 groups according to treatment strategy. The first group comprised lesions treated with DCB alone, the second group comprised lesions treated with POBA alone, and the third group comprised lesions undergoing PCI with DES. Lesions treated with a hybrid approach of DCB and DES were included in the DES group. The primary outcome of this study was TLR within a 5‐year follow‐up. Supplementary analyses were conducted for the 1‐year and 3‐year follow‐ups, and landmark analyses were conducted at 1 to 5 years and 3 to 5 years. TLR was defined as a repeat revascularization with PCI in the same lesion or coronary artery bypass graft surgery. Data on TLR were collected from the SCAAR. Secondary outcomes included all‐cause death, cardiovascular death, myocardial infarction, and any PCI. Supplementary analyses were also conducted on bleeding events, definite stent thrombosis, and coronary artery bypass surgery. Censorship dates and death status were ascertained by the National Board of Health and Welfare by deterministic linkage to the National Population Registry. Myocardial infarction was defined in accordance with the fourth universal definition of myocardial infarction (*International Classification of Diseases*, *Tenth Revision* [*ICD‐10*]: I21–I22) and data on myocardial infarction was collected through new registrations in the SCAAR. Cardiovascular death was ascertained up to July 2, 2021. All other outcomes were ascertained up to January 15, 2022, with complete follow‐up for all patients. Patients who died were censored in the analysis and were not considered to be at risk for any of the outcomes after death.

### Statistical Analysis

Continuous data are presented as means with SD. Normally distributed continuous variables were assessed using 1‐way ANOVA. Categorical data are presented as counts with percentages, and differences between categorical variables were studied using the χ^2^ test. TLR was analyzed on the lesion level, and each lesion was treated as a unique observation. Considering this assumption, we used a multilevel hierarchical Cox proportional hazard model with lesions nested on the patient level to reduce bias. After the lesion‐level analysis, the data were converted to patient‐level data to study all‐cause death, cardiovascular death, myocardial infarction, and any PCI. Outcome was also analyzed, stratified on type of ISR (DES‐ISR and BMS‐ISR). Outcomes were assessed using Kaplan–Meier estimates and univariable and multivariable Cox proportional hazards models. In the multivariable Cox proportional hazard analysis, we adjusted for inclusion year, age, sex, smoking status, diabetes, hypertension, hyperlipidemia, previous heart failure, renal failure, previous myocardial infarction, previous coronary artery bypass graft surgery, indication, number of lesions with ISR, lesion location, use of intravascular ultrasound or optic coherence tomography, American College of Cardiology/American Heart Association lesion classification, ISR‐type (DES‐ISR versus BMS‐ISR), stent layer (single versus multiple), and time to ISR (early ISR [<31 days] versus late ISR [31–365 days] versus very late ISR [>365 days]). We assessed if the Cox proportional hazards assumption was met for all the primary analyses. If the proportional hazards assumption was not met, Poisson regression was used to assess outcome using the same covariates as in the Cox proportional hazards models. Subgroup analyses were carried out for sex, age, diabetes, type of ISR (BMS‐ISR versus DES‐ISR), stent layer (single versus multiple), and indication (acute coronary syndrome versus stable coronary artery disease). TLR was used to study subgroups, and the analysis was carried out using the multivariable Cox proportional hazards models and results are presented along with interactive *P* values. A supplementary subgroup analysis was conducted on all‐cause death. Three sensitivity analyses were conducted. First, a multivariable Cox proportional hazard analysis was conducted including only variables with no missing values. Second, the 5‐year event rate of TLR was investigated for different types of DCBs. Third, an analysis excluding patients with multiple‐lesion PCI was carried out. In a separate analysis, BMS‐ISR was compared with DES‐ISR. Results from all Cox proportional hazards analyses are presented with hazard ratio (HR) and 95% CI along with *P* value. Results from all Poisson regression models are presented with risk ratio (RR) and 95% CI along with *P* value. All analyses were conducted on complete case data. A 2‐sided *P* value <0.05 was considered statistically significant. Data management and all statistical analyses were done in STATA SE version 17.0 (StataCorp, College Station, TX).

## Results

### Study Population Characteristics

For this study, 10 561 lesions from 9062 patients were included. The mean age was 69.6 years, and 78.2% of the patients were men. On the lesion level, 3322 (31.5%) of the lesions were treated with DCBs, 6096 (57.7%) with DESs, and 1143 (10.8%) with POBA. The proportion of missing data in the variables of interest were low; the variables smoking status and hyperlipidemia had 5.6% and 0.5% missing values, respectively (Table 1).

**Table 1:** Baseline Characteristics

Patient level	DCB (n=2752)	DES (n=5463)	POBA (n=847)	*P* value	Missing (%)
Inclusion time, n (%)					
2013–2015	733 (26.6)	1470 (26.9)	257 (30.3)	0.049	0.0
2016–2018	950 (34.5)	1995 (36.5)	293 (34.6)		
2019–2022	1069 (38.8)	1998 (36.6)	297 (35.1)		
Age, y, mean±SD	69.0±10.5	69.9±9.9	70.1±10.5	<0.001	0.0
Age≥80 y, n (%)	430 (15.6)	915 (16.7)	167 (19.7)	0.020	0.0
Sex, male, n (%)	2122 (77.1)	4303 (78.8)	661 (78.0)	0.23	0.0
Smoking status, n (%)					
Nonsmoker	1083 (41.1)	1873 (36.5)	307 (38.6)	<0.001	5.6
Previous smoker	1223 (46.4)	2466 (48.1)	365 (45.9)		
Active smoker	327 (12.4)	790 (15.4)	124 (15.6)		
Comorbidities					
Diabetes, n (%)	1082 (39.3)	1999 (36.6)	314 (37.1)	0.053	0.0
Hypertension, n (%)	1925 (69.9)	3598 (65.9)	553 (65.3)	<0.001	0.0
Hyperlipidemia, n (%)	2615 (95.4)	5061 (93.1)	758 (90.2)	<0.001	0.5
Previous heart failure, n (%)	555 (20.2)	958 (17.5)	180 (21.3)	0.002	0.0
Renal failure, n (%)	271 (9.8)	442 (8.1)	67 (7.9)	0.021	0.0
Estimated glomerular filtration rate, mean±SD	84.5±30.1	84.0±29.3	83.3±30.1	0.66	21.9
Previous stroke, n (%)	256 (9.3)	494 (9.0)	78 (9.2)	0.93	0.0
Previous myocardial infarction, n (%)	1904 (69.2)	3904 (71.5)	575 (67.9)	0.024	0.0
Previous coronary artery bypass graft surgery, n (%)	464 (16.9)	907 (16.6)	125 (14.8)	0.34	0.0
Procedural characteristics					
Contrast volume, mL, mean±SD	144.8±64.6	166.6±77.2	143.5±69.0	<0.001	0.0
Arterial access, n (%)					
Radial	2105 (76.5)	4154 (76.0)	610 (72.0)	0.032	0.0
Femoral	593 (21.5)	1172 (21.5)	218 (25.7)		
Other	54 (2.0)	137 (2.5)	19 (2.2)		
Indication, n (%)					
Stable coronary artery disease	859 (31.2)	1378 (25.2)	138 (16.3)	<0.001	0.0
Unstable angina	650 (23.6)	1070 (19.6)	129 (15.2)		
NSTEMI	806 (29.3)	1616 (29.6)	191 (22.6)		
STEMI	299 (10.9)	1110 (20.3)	297 (35.1)		
Other	138 (5.0)	289 (5.3)	92 (10.9)		
Number of ISRs, n (%)					
1	2423 (88.0)	4586 (83.9)	749 (88.4)	<0.001	0.0
2	296 (10.8)	754 (13.8)	80 (9.4)		
≥3	33 (1.2)	123 (2.3)	18 (2.1)		
Discharge medications, n (%)					
Acetylsalicylic acid	2249 (81.7)	4490 (82.2)	672 (79.3)	0.13	
P2Y_12_ inhibitor	2554 (92.8)	5091 (93.2)	743 (87.7)	<0.001	
Statin	2318 (84.2)	4653 (85.2)	711 (83.9)	0.42	
β blocker	2112 (76.7)	4326 (79.2)	640 (75.6)	0.007	
ACEi/ARB	1930 (70.1)	3860 (70.7)	597 (70.5)	0.89	
Calcium channel blocker	839 (30.5)	1690 (30.9)	239 (28.2)	0.28	
Lesion level					

	DCB (n=3322)	DES (n=6096)	POBA (n=1143)	*P* value	Missing (%)
Lesion characteristics					
Lesion location, n (%)					
Left main lesion	137 (4.1)	248 (4.1)	54 (4.7)	0.009	0.0
Other proximal lesion	1186 (35.7)	2396 (39.3)	443 (38.8)		
Distal lesion	1999 (60.2)	3452 (56.6)	646 (56.5)		
ACC/AHA lesion classification, n (%)					
Type A	219 (6.6)	250 (4.1)	80 (7.0)	<0.001	0.0
Type B1–B2	2115 (63.7)	3557 (58.3)	613 (53.6)		
Type C or B1–B2 with bifurcation	988 (29.7)	2289 (37.5)	450 (39.4)		
Stent or balloon diameter, mean±SD	3.1±0.5	3.3±0.6	3.1±0.7	<0.001	10.7
Use of IVUS or OCT, n (%)	466 (14.0)	908 (14.9)	263 (23.0)	<0.001	0.0
Rotablator	5 (0.2)	26 (0.4)	0 (0.0)	0.009	0.0
Shockwave	11 (0.3)	41 (0.7)	4 (0.3)	0.063	0.0
Cutting balloon	178 (5.4)	175 (2.9)	8 (0.7)	<0.001	0.0
Previous stent characteristics					
Stent layer, n (%)					
Single	3107 (93.5)	5812 (95.3)	1091 (95.5)	<0.001	0.0
Multiple	215 (6.5)	284 (4.7)	52 (4.5)		
ISR type, n (%)					
BMS‐ISR	645 (19.4)	2248 (36.9)	154 (13.5)	<0.001	0.0
DES‐ISR	2671 (80.4)	3831 (62.8)	986 (86.3)		
Other	6 (0.2)	17 (0.3)	3 (0.3)		
Diameter, mm, mean±SD	3.0±0.5	3.1±0.5	3.1±0.6	<0.001	3.4
Stent length, mm, mean±SD	22.3±8.6	20.6±8.1	22.7±8.8	<0.001	3.6
Time from stenting to ISR, n (%)					
Early ISR (≤31 d)	35 (1.1)	174 (2.9)	285 (24.9)	<0.001	
Late ISR (32–365 d)	784 (23.6)	824 (13.5)	244 (21.3)		
Very late ISR (>365 d)	2503 (75.3)	5098 (83.6)	614 (53.7)		

ACC/AHA indicates American College of Cardiology/American Heart Association; ACEi/ARB, angiotensin converting enzyme inhibitor/angiotensin receptor blocker; BMS, bare‐metal stent; DCB, drug‐coated balloon; DES, drug‐eluting stent; ISR, in‐stent restenosis; IVUS, intravascular ultrasound; NSTEMI, non–ST‐segment–elevation myocardial infarction; OCT, optical coherence tomography; POBA, plain old balloon angioplasty; and STEMI, ST‐segment–elevation myocardial infarction.

### DCBs Versus POBA

**Figure 2:**
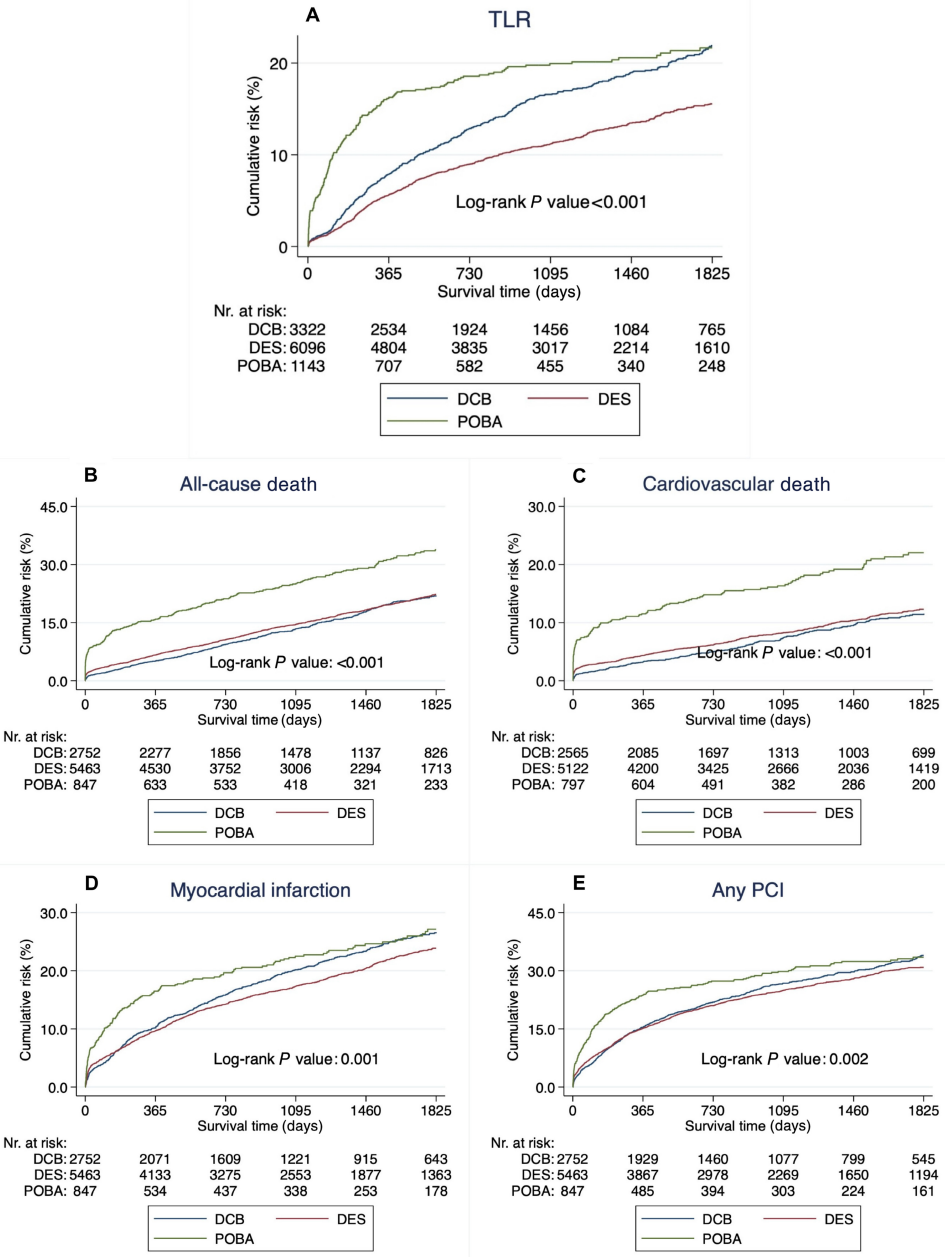
Outcome. Kaplan–Meier curves illustrating the event rate of (**A**) TLR, (**B**) all‐cause death, (**C**) cardiovascular death, (**D**) myocardial infarction, and (**E**) any PCI. DCB indicates drug‐coated balloon; DES, drug‐eluting stent; PCI, percutaneous coronary intervention; POBA, plain old balloon angioplasty; and TLR, target‐lesion revascularization.

The use of DCB angioplasty compared with POBA was associated with a lower RR of TLR (adjusted RR, 0.69 [95% CI, 0.57–0.82]; *P*<0.001) and all‐cause death (adjusted RR, 0.72 [95% CI, 0.59–0.88]; *P*=0.001). The use of DCB angioplasty was also associated with lower rates of cardiovascular death (adjusted HR, 0.59 [95% CI, 0.44–0.77]; *P*<0.001). No difference was observed for myocardial infarction (adjusted RR, 1.04 [95% CI, 0.85–1.27]; *P*=0.728) and any PCI (adjusted RR, 0.85 [95% CI, 0.72–1.01]; *P*=0.058) (Table 2 and Figure 2).

**Table 2:** Five‐Year Outcome

	Events (KM, %)			Unadjusted HR (95% CI); *P* value		Adjusted HR (95% CI)*	
	DCB	DES	POBA	DCB vs DES	DCB vs POBA	DCB vs DES	DCB vs POBA
TLR	416 (21.9)	548 (15.5)	165 (21.7)	1.45 (1.29–1.64); <0.001	0.77 (0.64–0.92); 0.005	1.20 (1.06–1.37); 0.005	0.69 (0.57–0.82); <0.001
All‐cause death	423 (22.0)	900 (22.3)	235 (33.8)	0.94 (0.84–1.06); 0.305	0.51 (0.44–0.60); <0.001	0.92 (0.81–1.04); 0.193	0.72 (0.59–0.88)†; 0.001
Cardiovascular death	198 (11.5)	451 (12.3)	139 (22.0)	0.88 (0.75–1.04); 0.141	0.41 (0.33–0.51); <0.001	0.84 (0.70–1.01); 0.062	0.59 (0.44–0.77); 0.003
Myocardial infarction	535 (26.6)	950 (23.9)	171 (27.1)	1.13 (1.01–1.25); 0.026	0.85 (0.72–1.01); 0.068	1.06 (0.95–1.19); 0.317	1.04 (0.85–1.27)†; 0.728
Any PCI	691 (34.0)	1290 (30.9)	223 (33.5)	1.07 (0.97–1.17); 0.164	0.83 (0.71–0.96); 0.013	0.97 (0.88–1.07); 0.486	0.85 (0.72–1.01)†; 0.058

ACC/AHA indicates American College of Cardiology/American Heart Association; DCB, drug‐coated balloon; DES, drug‐eluting stent; HR, hazard ratio; ISR, in‐stent restenosis; IVUS, intravascular ultrasound; KM, Kaplan–Meier estimates; OCT, optic coherence tomography; PCI, percutaneous coronary intervention; POBA, plain old balloon angioplasty; and TLR, target‐lesion revascularization.

* Adjusted for inclusion year, age, sex, smoking status, diabetes, hypertension, hyperlipidemia, previous heart failure, renal failure, previous myocardial infarction, previous coronary artery bypass graft surgery, indication, number of lesions with ISR, lesion location, use of IVUS or OCT, ACC/AHA lesion classification, ISR type (DES‐ISR vs BMS‐ISR), number of previous stents in target lesion (single vs multiple) and time to ISR (early ISR [<31 days] vs late ISR [31–365 days] vs very late ISR [>365 days]).

^†^ Outcome was assessed using Poisson regression, as the proportional hazard assumption was not met. Results are presented as risk ratios along with 95% CI.

**Figure 3:**
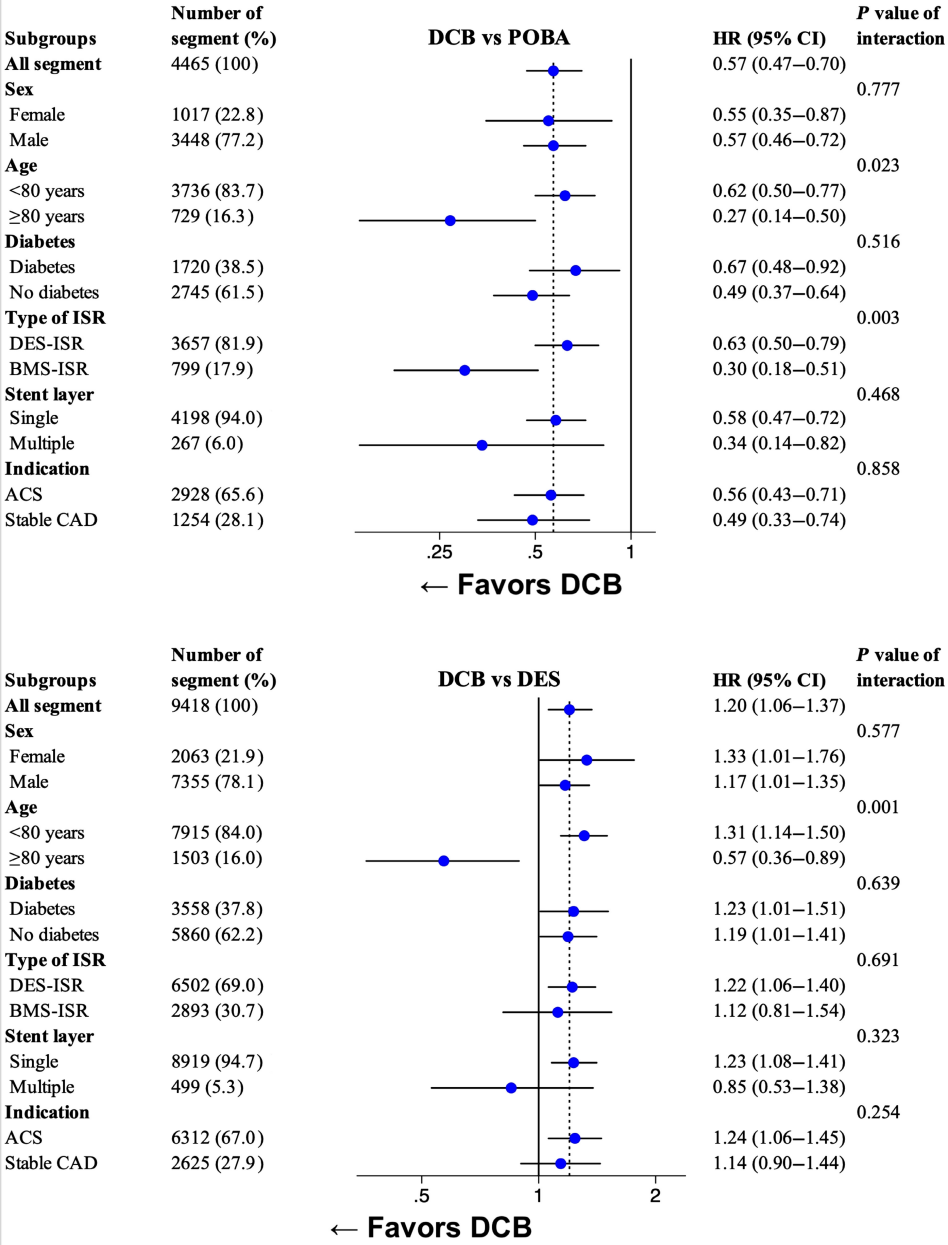
Subgroup analysis. Forest plot for subgroup analysis. The subgroups were analyzed on TLR using an adjusted* Cox proportional hazard model. *Adjusted for inclusion year, age, sex, smoking status, diabetes, hypertension, hyperlipidemia, previous heart failure, renal failure, previous myocardial infarction, previous coronary artery bypass graft surgery, indication, number of lesions with ISR, lesion location, use of IVUS or OCT, ACC/AHA lesion classification, ISR‐type (DES‐ISR vs BMS‐ISR), number of previous stents in target lesion (single vs multiple) and time to ISR (Early ISR [<31 days] vs late ISR [31–365 days] vs very late ISR [>365 days]). ACC/AHA indicates American College of Cardiology/American Heart Association; ACS, acute coronary syndrome; BMS, bare‐metal stent; CAD, coronary artery disease; DCB, drug‐coated balloon; DES, drug‐eluting stent; ISR, in‐stent restenosis; IVUS, intravascular ultrasound; OCT, optical coherence tomography; POBA, plain old balloon angioplasty; and TLR, target lesion revascularization.

**Figure 4:**
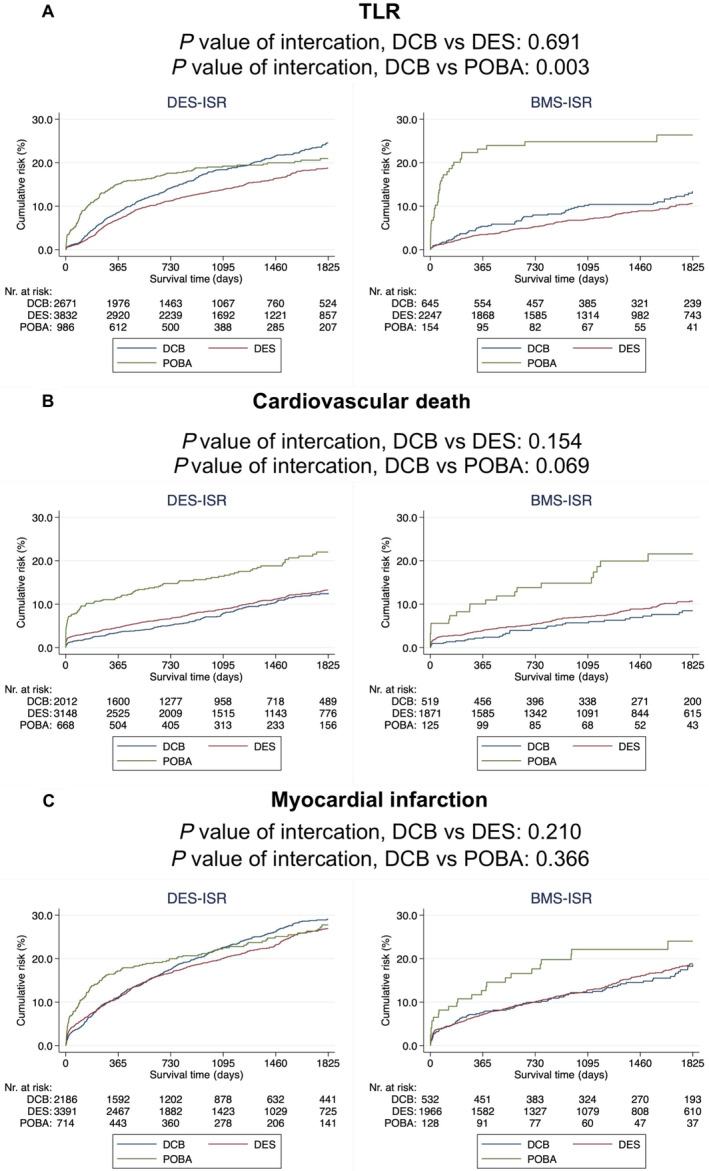
DES‐ISR and BMS‐ISR stratification. Kaplan–Meier curves illustrating the event rate of (**A**) TLR, (**B**) cardiovascular death, and (**C**) myocardial infarction. *P* value of interaction was also calculated using an adjusted* Cox proportional hazard model. *Adjusted for: inclusion year, age, sex, smoking status, diabetes mellitus, hypertension, hyperlipidemia, previous heart failure, renal failure, previous myocardial infarction, previous coronary artery by‐pass graft surgery, indication, number of lesions with ISR, lesion location, use of IVUS or OCT, ACC/AHA lesion classification, ISR type (DES‐ISR vs BMS‐ISR), number of previous stents in target lesion (single vs multiple) and time to ISR (Early ISR [<31 days] vs late ISR [31–365 days] vs very late ISR [>365 days]). ACC/AHA indicates American College of Cardiology/American Heart Association; BMS, bare‐metal stent; CAD, coronary artery disease; DCB, drug‐coated balloon; DES, drug‐eluting stent; HR, hazard ratio; ISR, in‐stent restenosis; POBA, plain old balloon angioplasty; and TLR, target lesion revascularization.

In the DCB versus POBA subgroup analysis of TLR, DCB angioplasty was more advantageous when used for patients ≥80 years (*P* value of interaction=0.023) and for BMS‐ISR (*P* value of interaction=0.003) (Figures 3 and 4). In the supplementary subgroup analysis investigating all‐cause death among the same subgroups, no subgroup interaction was observed (Figure S1). When stratifying on ISR type (DES‐ISR and BMS‐ISR), no subgroup interaction between DCB angioplasty and POBA was observed in terms of cardiovascular death and myocardial infarction (Figure 4).

### DCBs Versus DESs

The use of DCBs compared with DESs was associated with higher rates of TLR (adjusted HR, 1.20 [95% CI, 1.06–1.37]; *P*=0.005). No difference was observed for all‐cause death (adjusted HR, 0.92 [95% CI, 0.81–1.04]; *P*=0.193), cardiovascular death (adjusted HR, 0.84 [95% CI, 0.70–1.01]; *P*=0.062), myocardial infarction (adjusted HR, 1.06 [95% CI, 0.95–1.19]; *P*=0.317), or any PCI (adjusted HR, 0.97 [95% CI, 0.88–1.07]; *P*=0.485) (Table 2 and Figure 2).

In the DCB versus DES subgroup analysis of TLR, DCB angioplasty was more advantageous when used for patients ≥80 years (*P* value of interaction=0.001) (Figure 3). In the supplementary subgroup analysis investigating all‐cause death among the same subgroups, no subgroup interaction was observed (Figure S1). When stratifying on ISR type (DES‐ISR and BMS‐ISR), no subgroup interaction between DCB and DES was observed in terms of TLR, cardiovascular death, and myocardial infarction (Figure 4).

### Sensitivity and Supplementary Analysis

A multivariable Cox proportional hazard model including only variables with no missing values was conducted, excluding the variables smoking status and hyperlipidemia from the adjusting model. The results from this analysis were in line with the main analysis (Table S1).

We also conducted a sensitivity analysis excluding patients undergoing multilesion PCI. This analysis comprised 1453 patients treated with DCBs, 2581 with DESs, and 431 with POBA. The results of this analysis were in line with the main analysis (Table S2).

The 1‐year and 3‐year follow‐up analyses were consistent with the main results, and the landmark analysis showed a similar outcome between the groups after 3 years (Tables S3 and S4).

In a supplementary analysis investigating the association of different types of DCBs on outcome, the 5‐year event rate of TLR was similar, 23.4% for Invatec In.Pact Falcon (Medtronic, Minneapolis, MN), 22.4% for Braun SeQuent Please (Braun, Hesse, Germany), 21.5% for Biotronik Pantera Lux (Berlin, Germany), and 26.3% for other types of DCBs, log‐rank *P*=0.906 (Figure S2).

DCB angioplasty was associated with lower rates of coronary artery bypass surgery compared with POBA (adjusted HR, 0.56 [95% CI, 0.36–0.86] *P*=0.008]), no difference was observed compared with DESs. No statistically significant difference was observed in bleeding events or definite stent thrombosis between the groups (Figure S3).

### DES‐ISR Versus BMS‐ISR

Compared with BMS‐ISR, DES‐ISR was associated with a higher rate of TLR (adjusted HR, 1.39 [95% CI, 1.19–1.62]; *P*<0.001), myocardial infarction (adjusted HR, 1.34 [95% CI, 1.18–1.52]; *P*<0.001) and any PCI (adjusted HR, 1.16 [95% CI, 1.04–1.30]; *P*=0.007). No difference was observed for all‐cause death or cardiovascular death (Table S5).

## Discussion

To the best of our knowledge, this is the first study on PCI of ISR with DCBs, DESs, or POBA that includes any type of underlying stent and DCB. We found that the use of DCB was associated with lower rates of repeat revascularization and better survival when compared with POBA. Compared with DESs, DCBs exhibited a higher rate of TLR while maintaining comparable survival rates.

Choosing treatment strategy for patients with ISR poses a complex challenge as multiple factors need to be considered, including the high risk of recurrent restenosis. Previous studies have shown that DES‐ISR is associated with higher rates of recurrent myocardial infarctions compared with BMS‐ISR.^11^ In the RIBS IV (Comparison of the Efficacy of Everolimus‐Eluting Stents Versus Drug‐Eluting Balloons in Patients With In‐Stent Restenosis) trial, which only included DES‐ISR, the 3‐year event rate of TLR with DCB was 15.6%.^20^ While the RIBS V trial included only patients with BMS‐ISR, the 3‐year event rate of TLR was 8% after DCB.^21^ In this study, which included real‐world patients with ISR, 70.9% presented with DES‐ISR, and the 3‐year event rate of TLR was 16.6% after DCB (Table 1 and Table S3). The results showed that DES‐ISR had a 39% increased rate of TLR, and a 34% higher rate of myocardial infarction compared with BMS‐ISR after 5 years from PCI (Table S5). The likely reason for this finding is that the DES‐ISR, due to a lower risk of ISR with DESs, represents a patient population with more severe cardiovascular disease and corresponding worse clinical outcome. It might be also plausible to consider that DESs are more prone to neoatherosclerosis and DES‐ISR represents the failure of an antiproliferative drug‐based therapy. Since a DCB is just a carrier and does not provide mechanical support by vessel scaffolding, DES‐ISR may be associated with a lower long‐term rate of success after treatment with DCBs compared with BMS‐ISR. The results of this study show superior outcome after PCI with both DCBs and DESs for BMS‐ISR compared with DES‐ISR.

Despite DES‐ISR having a higher risk of treatment failures, the subgroup analysis indicated no discernible difference between BMS‐ISR and DES‐ISR in terms of TLR, all‐cause death, cardiovascular death, and myocardial infarction when comparing the treatment with DCBs versus DESs (Figures 3 and 4; Figure S1). Most randomized trials comparing DCBs with DESs for ISR have focused either on BMS‐ISR or DES‐ISR and used a specific type of DCB restricting the generalizability of these studies. In this study, we aimed to assess generalizable results including any underlying stent and different types of DCBs for all‐comer patients with ISR.

Within the first year, DCB angioplasty appears to be substantially more beneficial compared with POBA. The 1‐year event rate of TLR in the present study shows 7.9% versus 16.3% (RR, 0.48) for DCB angioplasty and POBA, respectively. The recently published AGENT IDE (A Clinical Trial to Assess the Agent Paclitaxel Coated PTCA Balloon Catheter for the Treatment of Subjects With In‐Stent Restenosis) trial showed a higher 1‐year event rate of TLR compared with the present study yet shows a similar risk ratio of TLR, 12.4% versus 24.0% (RR, 0.52).^22^ Despite an early benefit, the long‐term efficacy of DCB angioplasty, as indicated by visually examining the Kaplan–Meier curves in Figure 2 and the landmark analysis (Table S4), needs to be noted. The long‐term outcome between DCB angioplasty and POBA in this study aligns with the landmark analysis in the ISAR DESIRE 3 (Coronary Artery Restenosis Treatment With Plain Balloon, Drug‐Coated Balloon, or Drug‐Eluting Stent) trial, which shows similar event rates of TLR after 1 year between DCB angioplasty and POBA.^23^ This becomes evident in the results of the subgroup analysis, indicating a better outcome when DCB angioplasty was used for older patients aged ≥80 years, possibly benefiting from the early benefits of DCB angioplasty due to shorter life expectancies (Figure 3).

A limitation of the AGENT IDE trial was the absence of a DES arm, which plausibly is the preferred strategy for patients with ISR. There are 3 randomized studies to date comparing DCBs with DESs, including a mixed population with ISR in either BMSs or DESs (the DARE [A Randomized Comparison of Paclitaxel‐Eluting Balloon Versus Everolimus‐Eluting Stent for the Treatment of Any In‐Stent Restenosis] trial, the RIBS IV/V trial, and the BIOLUX‐RCT [Angiographic and Clinical Performance of a Paclitaxel‐Coated Balloon Compared to a Second‐Generation Sirolimus‐Eluting Stent in Patients With In‐Stent Restenosis] trial).^14^, ^17^, ^24^ Most previous randomized trials have small study populations and are underpowered to prove any significant difference in clinical outcomes. The size of the current study population allows enough power to assess clinical outcomes such as TLR and death. In this study, DCBs were associated with a 20% increased rate of TLR when compared with DESs. This is in line with the DAEADALUS (Paclitaxel‐Coated Balloon Angioplasty Versus Drug‐Eluting Stenting for the Treatment of Coronary In‐Stent Restenosis) meta‐analysis of 10 randomized trials showing higher rates of TLR after 3 years when a DCB was used (HR, 1.32 [95% CI, 1.02–1.70]).^11^ However, results among previous randomized trials vary, and it becomes challenging to draw firm conclusions on treatment efficacies. Furthermore, the preceding studies are limited to short follow‐up times, ranging from 1 to 3 years, and differences between interventions can require longer observational times to emerge. In this study, the Kaplan–Meier estimates showed a larger difference between DCBs and DESs for TLR with longer follow‐up time, which underscores the necessity of longer follow‐up times for future randomized trials assessing DCBs for ISR.

### Limitations

This study has several limitations. Despite our best attempts to account for confounding, underlying anatomic and clinical selection bias cannot be ruled out. Treatment assignment was not random. The SCAAR lacks information on why a particular lesion/patient was selected for a DCB, DES, or POBA. Decisions may be clinically valid and based on factors not captured in the registry. The Kaplan–Meier curves for all‐cause death and cardiovascular death separates very early for POBA. The reasons for this might be due to confounding, and results for survival after POBA should be interpreted carefully. It is possible that POBA was more frequently used in older, frailer, and comorbid patients, and despite adjustment, residual confounding effects have continued to affect the results. Against current recommendations, the use of intracoronary imaging was low in this study. The low intracoronary imaging penetration could have affected stent/balloon optimization. Despite low use of intracoronary imaging, this reflects real‐world practice. The SCAAR lacks information on some important lesion‐level angiographic characteristics, such as minimum lumen diameter, percentage diameter stenosis, and so on, procedural aspects, such as predilation success, bailout stenting, and the like.

## Conclusions

In this long‐term nationwide analysis, our findings indicate lower rates of TLR for DCB‐treated lesions compared with POBA‐treated lesions. While DESs demonstrated lower TLR rates than DCBs, the lack of mortality differences indicates that DCBs are a viable option, particularly for patients for whom a DES might not be suitable, but that DESs should be considered as the first‐line strategy when assessing ISR.

## Sources of Funding

This work was supported by The Swedish Heart and Lung Foundation; ALF; Skane University Hospital funds; Märta Winkler Foundation; Thorsten Westerströms Foundation; Sven‐Eric Lundgren Foundation for Medical Research; the Crafoord Foundation; and the Swedish Medical Association. The sponsors were not involved in the study design, collection of data, analysis of data, interpretation of data, writing of the manuscript, approving the manuscript, or the decision to submit the manuscript for publication. The decision to submit the manuscript was solely the authors’.

## Disclosures

None.

## Supporting information

Tables S1–S5
Figures S1–S3
