# Supplementary material for: Drug‐Coated Balloons Versus Drug‐Eluting Stents or Plain Old Balloon Angioplasty: A Long‐Term in‐Stent Restenosis Study
Source: J Am Heart Assoc. 2024 Nov 22;13(23):e036839. doi: 10.1161/JAHA.124.036839 (PMC11681599; doi:10.1161/JAHA.124.036839)
Supplement: Supplementary file 1 — Tables S1–S5 Figures S1–S3 [file JAH3-13-e036839-s001.pdf]

Supplemental Material

Table S1. Adjusting for variables with no missing values

	Adjusted HR, (95% CI) *	
	**DCB vs DES**	**DCB vs POBA**
TLR	1.22 (1.07-1.38)<br>p-value: 0.002	0.58 (0.47-0.70)<br>p-value: <0.001
All-cause mortality	0.95 (0.84-1.07)<br>p-value: 0.379	0.66 (0.55-0.79)<br>p-value: <0.001
Cardiovascular mortality	0.87 (0.73-1.04)<br>p-value: 0.127	0.59 (0.46-0.77)<br>p-value: <0.001
Myocardial infarction	1.06 (0.95-1.19)<br>p-value: 0.292	0.93 (0.76-1.13)<br>p-value: 0.444
Any PCI	0.98 (0.89-1.07)<br>p-value: 0.624	0.74 (0.62-0.87)<br>p-value: <0.001

*Adjusted for: inclusion year, age, sex, diabetes mellitus, hypertension, previous heart failure, renal failure, previous myocardial infarction, previous coronary artery by-pass graft surgery, indication, number of lesions with ISR, lesion location, use of IVUS or OCT, ACC/AHA lesion classification, ISR-type (DES-ISR vs BMS-ISR), number of previous stents in target lesion (single vs multiple) and time to ISR (Early ISR [<31 days] vs late ISR [31-365 days] vs very late ISR [>365 days]).

ACC/AHA = American College of Cardiology/American Heart Association; CI = confidence interval; DCB = drug-coated balloon; DES =drug-eluting stent; HR = hazard ratio; ISR = in-stent restenosis; IVUS = intravascular ultrasound; OCT = optic coherence tomography; PCI = percutaneous coronary intervention; POBA = plain old balloon angioplasty; TLR = target lesion revascularization.

Table S2. Exclusion of multiple-lesion PCI patients

	Events, (KM, %)			Unadjusted HR, (95% CI)		Adjusted HR, (95% CI)*	
	**DCB**	**DES**	**POBA**	**DCB vs DES**	**DCB vs POBA**	**DCB vs DES**	**DCB vs POBA**
TLR	268 (24.7%)	320 (17.4%)	95 (27.1%)	1.55 (1.32-1.82) p-value: <0.001	0.73 (0.57-0.93) p-value: 0.012	1.26 (1.06-1.51) p-value: 0.010	0.50 (0.37-0.67) p-value: <0.001
All-cause mortality	228 (22.5%)	438 (23.0%)	115 (32.2%)	0.94 (0.79-1.08) p-value: 0.335	0.58 (0.46-0.72) p-value: <0.001	0.96 (0.80-1.14) p-value: 0.611	0.73 (0.55-0.96) p-value: 0.026
Cardiovascular mortality	105 (11.5%)	210 (12.4%)	72 (22.3%)	0.89 (0.70-1.13) p-value: 0.330	0.43 (0.32-0.58) p-value: <0.001	0.89 (0.69-1.15) p-value: 0.384	0.62 (0.42-0.91) p-value: 0.015
Myocardial infarction	316 (29.3%)	474 (25.6%)	84 (26.1%)	1.20 (1.04-1.38) p-value: 0.012	1.04 (0.81-1.32) p-value: 0.769	1.13 (0.97-1.31) p-value: 0.130	1.21 (0.91-1.62) p-value: 0.196
Any PCI	393 (35.9%)	598 (30.6%)	122 (34.5%)	1.18 (1.04-1.34) p-value: 0.010	0.84 (0.69-1.03) p-value: 0.103	1.05 (0.92-1.20) p-value: 0.484	0.69 (0.55-0.88) p-value: 0.003

In this analysis all patient undergoing multiple-lesion PCI were excluded. The analysis comprised 1453 patients treated with DCB, 2581 with DES and 431 with POBA.

*Adjusted for: inclusion year, age, sex, smoking status, diabetes mellitus, hypertension, hyperlipidemia, previous heart failure, renal failure, previous myocardial infarction, previous coronary artery by-pass graft surgery, indication, number of lesions with ISR, lesion location, use of IVUS or OCT, ACC/AHA lesion classification, ISR-type (DES-ISR vs BMS-ISR), number of previous stents in target lesion (single vs multiple) and time to ISR (Early ISR [<31 days] vs late ISR [31-365 days] vs very late ISR [>365 days]).

ACC/AHA = American College of Cardiology/American Heart Association; CI = confidence interval; DCB = drug-coated balloon; DES = drug-eluting stent; HR = hazard ratio; ISR = in-stent restenosis; IVUS = intravascular ultrasound; KM = Kaplan-Meier estimates; OCT = optic coherence tomography; PCI = percutaneous coronary intervention; PCI = percutaneous coronary intervention; POBA = plain old balloon angioplasty; TLR = target lesion revascularization.

Table S3. 1-year and 3-year follow-up

	Events, (KM, %)			Unadjusted HR, (95% CI)		Adjusted HR, (95% CI) *	
	**DCB**	**DES**	**POBA**	**DCB vs DES**	**DCB vs POBA**	**DCB vs DES**	**DCB vs POBA**
**1-year**							
TLR	238 (7.9%)	311 (5.6%)	161 (16.3%)	1.45 (1.27-1.66) p-value: <0.001	0.43 (0.35-0.54) p-value: <0.001	1.17 (1.02-1.34) p-value: 0.028	0.31 (0.25-0.40) p-value: <0.001
All-cause mortality	134 (5.1%)	350 (6.7%)	132 (15.9%)	0.75 (0.62-0.92) p-value: 0.006	0.29 (0.23-0.37) p-value: <0.001	0.81 (0.65-1.01) p-value: 0.064	0.45 (0.33-0.62) p-value: <0.001
Cardiovascular mortality	79 (3.3%)	215 (4.4%)	89 (11.5%)	0.73 (0.57-0.95) p-value: 0.018	0.26 (0.19-0.35) p-value: <0.001	0.78 (0.59-1.05) p-value: 0.101	0.48 (0.32-0.71) p-value: <0.001
Myocardial infarction	262 (10.2%)	493 (9.7%)	123 (16.5%)	1.04 (0.90-1.21) p-value: 0.588	0.58 (0.46-0.71) p-value: <0.001	0.96 (0.82-1.13) p-value: 0.648	0.62 (0.48-0.79) p-value: <0.001
Any PCI	394 (15.6%)	768 (15.1%)	177 (23.8%)	1.00 (0.89-1.13) p-value: 0.968	0.59 (0.49-0.70) p-value: <0.001	0.93 (0.82-1.06) p-value: 0.270	0.51 (0.41-0.62) p-value: <0.001
**3-year**							
TLR	436 (16.6%)	554 (11.2%)	188 (19.9%)	1.45 (1.27-1.65) p-value: <0.001	0.68 (0.57-0.82) p-value: <0.001	1.17 (1.02-1.34) p-value: 0.028	0.50 (0.41-0.61) p-value: <0.001
All-cause mortality	304 (13.4%)	681 (14.5%)	194 (25.0%)	0.89 (0.78-1.02) p-value: 0.088	0.45 (0.38-0.54) p-value: <0.001	0.89 (0.76-1.03) p-value: 0.108	0.57 (0.45-0.71) p-value: <0.001
Cardiovascular mortality	152 (7.5%)	359 (8.3%)	118 (16.3%)	0.85 (0.70-1.03) p-value: 0.088	0.37 (0.29-0.47) p-value: <0.001	0.83 (0.67-1.02) p-value: 0.073	0.56 (0.41-0.76) p-value: <0.001
Myocardial infarction	457 (20.2%)	794 (17.4%)	156 (22.5%)	1.15 (1.02-1.28) p-value: 0.021	0.79 (0.66-0.95) p-value: 0.013	1.06 (0.94-1.20) p-value: 0.325	0.86 (0.69-1.06) p-value: 0.160
Any PCI	612 (26.8%)	1149 (24.9%)	210 (29.8%)	1.05 (0.96-1.16) p-value: 0.282	0.77 (0.66-0.90) p-value: 0.001	0.95 (0.85-1.05) p-value: 0.310	0.67 (0.56-0.80) p-value: <0.001

*Adjusted for: inclusion year, age, sex, smoking status, diabetes mellitus, hypertension, hyperlipidemia, previous heart failure, renal failure, previous myocardial infarction, previous coronary artery by-pass graft surgery, indication, number of lesions with ISR, lesion location, use of IVUS or OCT, ACC/AHA lesion classification, ISR-type (DES-ISR vs BMS-ISR), number of previous stents in target lesion (single vs multiple) and time to ISR (Early ISR [<31 days] vs late ISR [31-365 days] vs very late ISR [>365 days]).

ACC/AHA = American College of Cardiology/American Heart Association; BMS = bare metal stent; CI = confidence interval; DCB = drug-coated balloon; DES = drug-eluting stent; HR = hazard ratio; ISR = in-stent restenosis; IVUS = intravascular ultrasound; KM = Kaplan-Meier estimates; OCT = optic coherence tomography; PCI = percutaneous coronary intervention; POBA = plain old balloon angioplasty; TLR = target lesion revascularization.

Table S4. Landmark analysis

	Events, (KM, %)			Unadjusted HR, (95% CI)		Adjusted HR, (95% CI) *	
	**DCB**	**DES**	**POBA**	**DCB vs DES**	**DCB vs POBA**	**DCB vs DES**	**DCB vs POBA**
**1-to-5 years**							
TLR	267<br>(15.2%)	357<br>(10.5%)	34<br>(6.4%)	1.45 (1.27-1.65)<br>p-value: <0.001	2.37 (1.65-3.41)<br>p-value: <0.001	1.17 (1.02-1.34)<br>p-value: 0.028	1.99 (1.32-3.00)<br>p-value: 0.001
All-cause mortality	289<br>(17.8%)	550<br>(16.8%)	103<br>(21.3%)	1.06 (0.92-1.22)<br>p-value: 0.406	0.80 (0.64-1.00)<br>p-value: 0.052	0.99 (0.85-1.16)<br>p-value: 0.894	0.81 (0.62-1.05)<br>p-value: 0.118
Cardiovascular mortality	119<br>(8.5%)	236<br>(8.3%)	50<br>(11.9%)	1.02 (0.82-1.27)<br>p-value: 0.859	0.68 (0.49-0.95)<br>p-value: 0.024	0.89 (0.70-1.12)<br>p-value: 0.319	0.73 (0.49-1.08)<br>p-value: 0.111
Myocardial infarction	273<br>(18.3%)	457<br>(15.7%)	48<br>(12.8%)	1.22 (1.05-1.42)<br>p-value: 0.008	1.56 (1.15-2.12)<br>p-value: 0.004	1.18 (1.00-1.38)<br>p-value: 0.046	1.77 (1.23-2.54)<br>p-value: 0.002
Any PCI	297<br>(21.9%)	522<br>(18.6%)	46<br>(12.7%)	1.17 (1.01-1.35)<br>p-value: 0.033	1.76 (0.29-2.40)<br>p-value: <0.001	1.02 (0.88-1.18)<br>p-value: 0.814	1.63 (1.15-2.34)<br>p-value: 0.007
**3-to-5 years**							
TLR	69<br>(6.4%)	114<br>(4.9%)	7<br>(2.1%)	1.45 (1.27-1.65)<br>p-value: <0.001	3.11 (1.42-6.82)<br>p-value: 0.005	1.17 (1.02-1.34)<br>p-value: <0.028	3.11 (1.20-8.01)<br>p-value: 0.019
All-cause mortality	119<br>(9.9%)	219<br>(9.1%)	41<br>(11.8%)	1.11 (0.89-1.38)<br>p-value: 0.375	0.82 (0.58-1.17)<br>p-value: 0.282	1.03 (0.81-1.32)<br>p-value: 0.784	0.91 (0.60-1.38)<br>p-value: 0.655
Cardiovascular mortality	46<br>(4.4%)	92<br>(4.4%)	21<br>(6.8%)	1.02 (0.71-1.45)<br>p-value: 0.928	0.62 (0.37-1.04)<br>p-value: 0.070	0.91 (0.62-1.33)<br>p-value: 0.612	0.73 (0.39-1.35)<br>p-value: 0.314
Myocardial infarction	78<br>(8.1%)	156<br>(7.9%)	15<br>(6.0%)	1.04 (0.79-1.36)<br>p-value: 0.782	1.46 (0.84-2.53)<br>p-value: 0.183	1.03 (0.77-1.37)<br>p-value: 0.861	1.65 (0.87-3.13)<br>p-value: 0.129
Any PCI	79<br>(9.8%)	141<br>(7.9%)	13<br>(5.2%)	1.17 (0.89-1.55)<br>p-value: 0.253	1.73 (0.96-3.11)<br>p-value: 0.067	1.13 (0.85-1.51)<br>p-value: 0.401	1.87 (0.94-3.69)<br>p-value: 0.073

*Adjusted for: inclusion year, age, sex, smoking status, diabetes mellitus, hypertension, hyperlipidemia, previous heart failure, renal failure, previous myocardial infarction, previous coronary artery by-pass graft surgery, indication, number of lesions with ISR, lesion location, use of IVUS or OCT, ACC/AHA lesion classification, ISR-type (DES-ISR vs BMS-ISR), number of previous stents in target lesion (single vs multiple) and time to ISR (Early ISR [<31 days] vs late ISR [31-365 days] vs very late ISR [>365 days]).

ACC/AHA = American College of Cardiology/American Heart Association; BMS = bare metal stent; CI = confidence interval; DCB = drug-coated balloon; DES = drug-eluting stent; HR = hazard ratio; ISR = in-stent restenosis; IVUS = intravascular ultrasound; KM = Kaplan-Meier estimates; OCT = optic coherence tomography; PCI = percutaneous coronary intervention; POBA = plain old balloon angioplasty; TLR = target lesion revascularization.

Table S5. DES-ISR vs BMS-ISR

	Events (KM, %)		HR, (95% CI)	
	**DES-ISR**	**BMS-ISR**	**Unadjusted**	**Adjusted***
TLR	1091 (21.1%)	275 (12.0%)	1.88 (1.64-2.16) p-value: <0.001	1.39 (1.19-1.62) p-value: <0.001
All-cause mortality	1116 (24.7%)	406 (19.8%)	1.30 (1.16-1.46) p-value: <0.001	1.09 (0.95-1.24) p-value: 0.215
Cardiovascular mortality	565 (14.0%)	206 (10.8%)	1.32 (1.12-1.54) p-value: 0.001	1.07 (0.89-1.29) p-value: 0.465
Myocardial infarction	1247 (27.7%)	380 (19.1%)	1.59 (1.42-1.78) p-value: <0.001	1.34 (1.18-1.52) p-value: <0.001
Any PCI	1617 (34.7%)	550 (26.3%)	1.39 (1.26-1.53) p-value: <0.001	1.16 (1.04-1.30) p-value: 0.007

*Adjusted for: inclusion year, age, sex, smoking status, diabetes mellitus, hypertension, hyperlipidemia, previous heart failure, renal failure, previous myocardial infarction, previous coronary artery by-pass graft surgery, indication, number of lesions with ISR, lesion location, use of IVUS or OCT, ACC/AHA lesion classification, treatment strategy (DCB, DES and POBA), number of previous stents in target lesion (single vs multiple) and time to ISR (Early ISR [<31 days] vs late ISR [31-365 days] vs very late ISR [>365 days]).

ACC/AHA = American College of Cardiology/American Heart Association; CI = confidence interval; BMS = bare metal stent; DCB = drug-coated balloon; DES = drug-eluting stent; HR = hazard ratio; ISR = in-stent restenosis; IVUS = intravascular ultrasound; KM = Kaplan-Meier estimates; OCT = optic coherence tomography; PCI = percutaneous coronary intervention; POBA = plain old balloon angioplasty; TLR = target lesion revascularization.

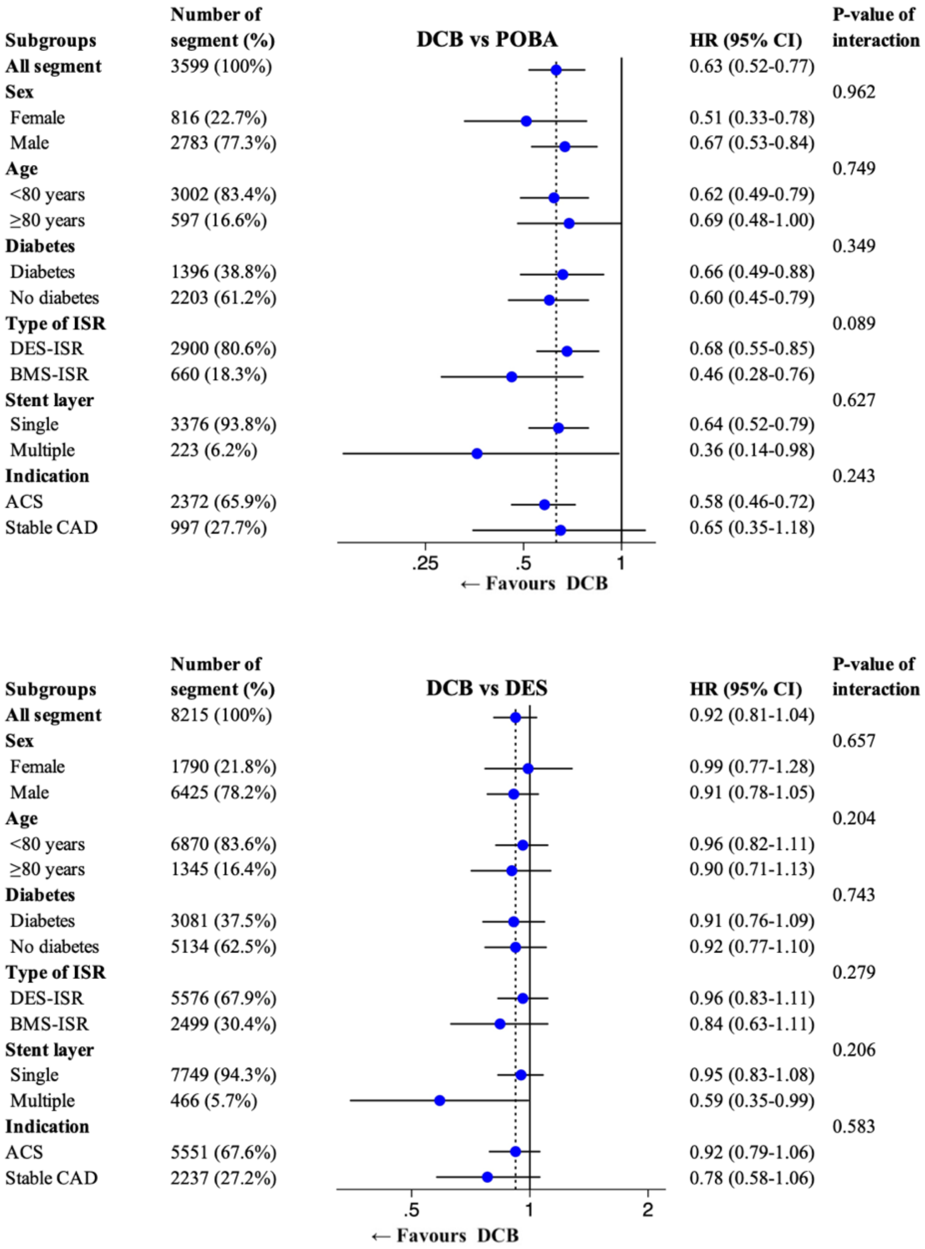

Figure S1. Subgroup analysis on all-cause mortality, Forest plot for subgroup analysis. The subgroups were analysed on all-cause mortality using an adjusted* Cox regression model. ACC/AHA = American College of Cardiology/American Heart Association; ACS = acute coronary syndrome; BMS = bare metal stent; CI = confidence interval; DCB = drug-coated balloon; DES = drug-eluting stent; HR = hazard ratio; ISR = in-stent restenosis; IVUS = intravascular ultrasound; OCT = optic coherence tomography; POBA = plain old balloon angioplasty.

*Adjusted for: inclusion year, age, sex, smoking status, diabetes mellitus, hypertension, hyperlipidemia, previous heart failure, renal failure, previous myocardial infarction, previous coronary artery by-pass graft surgery, indication, number of lesions with ISR, lesion location, use of IVUS or OCT, ACC/AHA lesion classification, ISR-type (DES-ISR vs BMS-ISR), number of previous stents in target lesion (single vs multiple) and time to ISR (Early ISR [<31 days] vs late ISR [31-365 days] vs very late ISR [>365 days]).

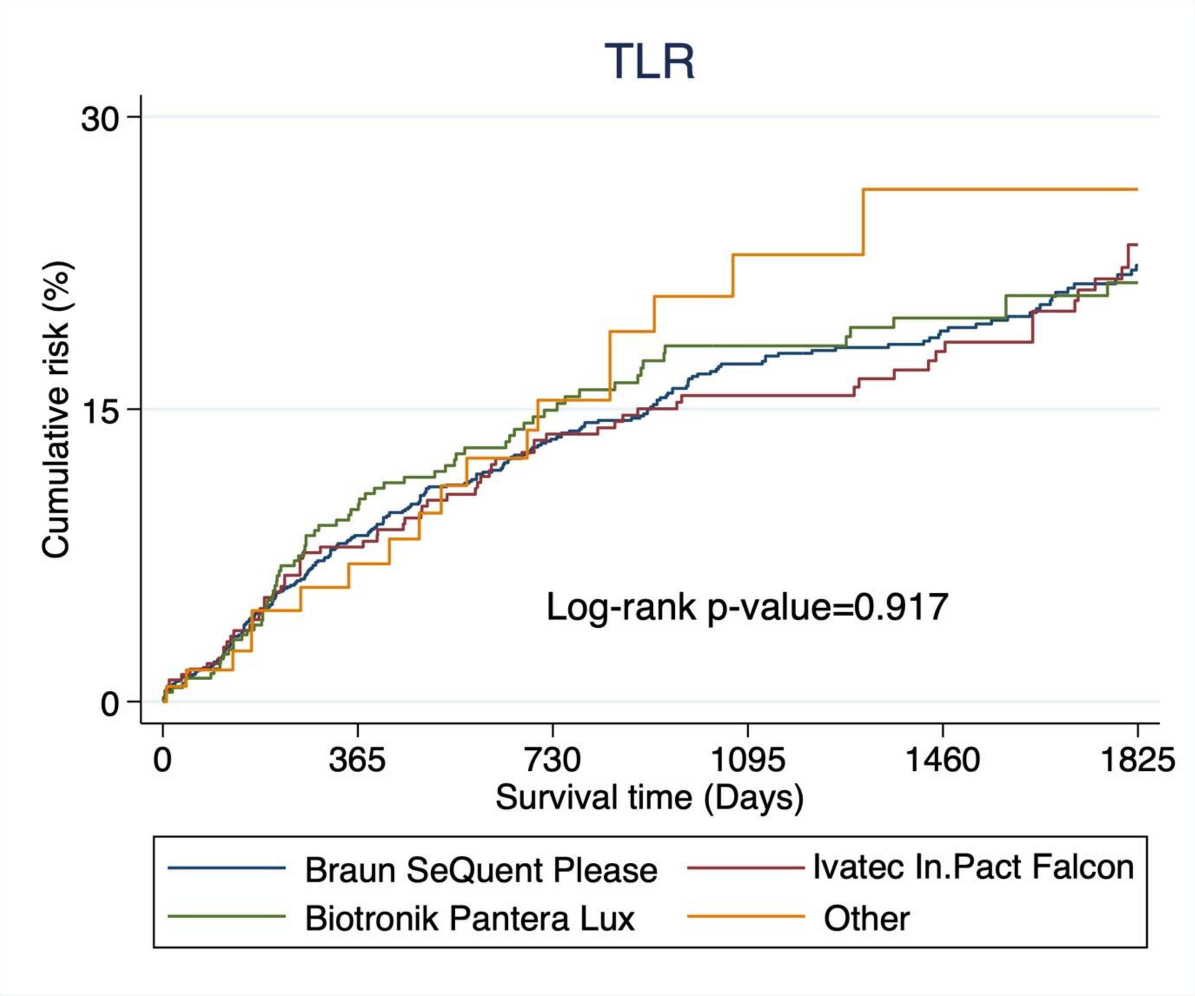

Figure S2. Different types of DCB, Time-to-event Kaplan Meier curve illustrating the event rate of TLR for different types of DCB. DCB = drug-coated balloon; TLR = target lesion revascularization.

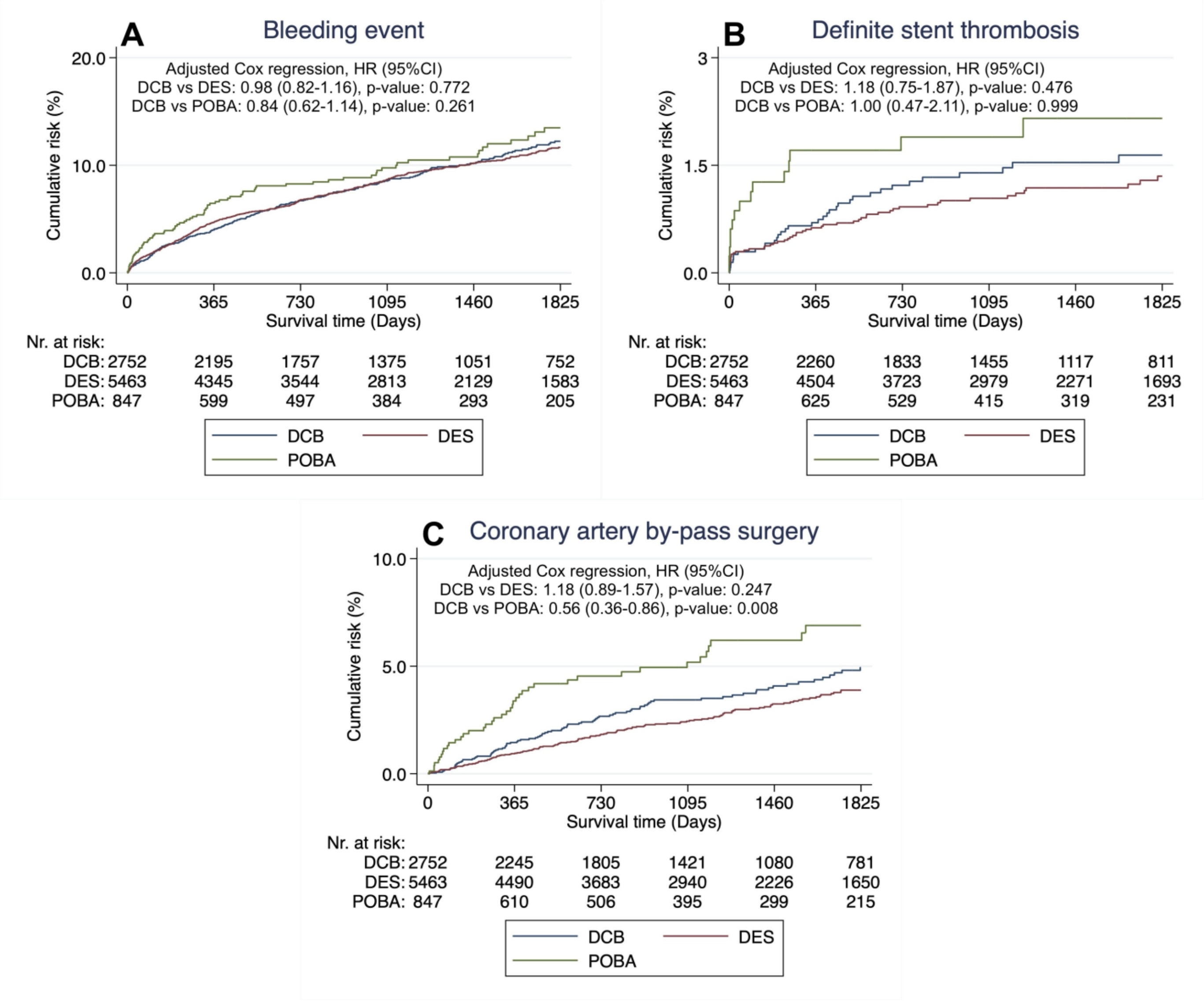

Figure S3. Bleeding event, definite stent thrombosis and coronary artery by-pass surgery, Time-to-event Kaplan Meier curves illustrating the event rate of (A) bleeding event, (B) definite stent thrombosis and (C) coronary artery by-pass graft surgery. In the Cox regression analysis we adjusted for: inclusion year, age, sex, smoking status, diabetes mellitus, hypertension, hyperlipidemia, previous heart failure, renal failure, previous myocardial infarction, previous coronary artery by-pass graft surgery, indication, number of lesions with ISR, lesion location, use of IVUS or OCT, ACC/AHA lesion classification, ISR-type (DES-ISR vs BMS-ISR), number of previous stents in target lesion (single vs multiple) and time to ISR (Early ISR [<31 days] vs late ISR [31-365 days] vs very late ISR [>365

days]). ACC/AHA = American College of Cardiology/American Heart Association; CI = confidence interval; BMS = bare metal stent; DCB = drug-coated balloon; DES = drug-eluting stent; HR = hazard ratio; ISR = in-stent restenosis; IVUS = intravascular ultrasound; OCT = optic coherence tomography; POBA = plain old balloon angioplasty.
